# What do parents think about parental participation in school-based interventions on energy balance-related behaviours? a qualitative study in 4 countries

**DOI:** 10.1186/1471-2458-11-881

**Published:** 2011-11-23

**Authors:** Wendy Van Lippevelde, Maïté Verloigne, Ilse De Bourdeaudhuij, Mona Bjelland, Nanna Lien, Juan M Fernández-Alvira, Luis A Moreno, Eva Kovacs, Johannes Brug, Lea Maes

**Affiliations:** 1 Department of Public Health, Ghent University, Watersportlaan 2, 9000 Ghent, Belgium; 2 Department of Movement and Sport Sciences, Ghent University, Watersportlaan 2, 9000 Ghent, Belgium; 3 Department of Nutrition, University of Oslo, P.O.Box 1046, Blindern 0316, Oslo, Norway; 4 Instituto Aragone' s de Ciencias de la Salud, University of Zaragoza, Edificio Cervantes Corona de Aragón, 42, 50009 Zaragoza, Spain; 5 Department of Paediatrics, University of Pécs, H-7623 Pécs, Hungary; 6 Department of Epidemiology and the EMGO Institute for Health and Care Research, VU University Medical Center, Van der Boechorststraat 7, 1081, BT Amsterdam, the Netherlands

## Abstract

**Background:**

Overweight and obesity in youth has increased dramatically. Therefore, overweight prevention initiatives should start early in life and target modifiable energy balance-related behaviours. Parental participation is often advocated as important for school-based interventions, however, getting parents involved in school-based interventions appears to be challenging based on earlier intervention experiences. The purpose of this study was to get insight into the determinants of and perspectives on parental participation in school-interventions on energy balance-related behaviours (physical activity, healthy eating, sedentary behaviours) in parents of ten- to twelve-year olds in order to develop an effective parental module for school-based interventions concerning energy balance-related behaviours.

**Methods:**

Four countries (Belgium, Hungary, Norway and Spain) conducted the focus group research based on a standardised protocol and a semi-structured questioning route. A variation in parental socio-economic status (SES) and parental school involvement was taken into account when recruiting the parents. The audio taped interviews were transcribed, and a qualitative content analysis of the transcripts was conducted in each country.

**Results:**

Seventeen focus group interviews were conducted with a total of 92 parents (12 men, 80 women). Physical activity was considered to be a joint responsibility of school and parents, nutrition as parent's responsibility but supported by the school, and prevention of sedentary behaviours as parent's sole responsibility. Parents proposed interactive and practical activities together with their child as the best way to involve them such as cooking, food tasting, nutrition workshops, walking or cycling tours, sport initiations together with their child. Activities should be cheap, on a convenient moment, focused on their children and not on themselves, not tutoring, not theoretical, and school-or home-based.

**Conclusions:**

Parents want to be involved in activities related to energy balance-related behaviours if this implies 'doing things together' with their child at school or at home.

## Background

Childhood overweight and obesity has increased dramatically during the last decades and is currently stabilizing in most developed countries [1,2]. Overweight is associated with different physical and psychosocial health problems in childhood and later life [3]. Therefore, overweight prevention initiatives promoting healthful diets and physical activity (PA) and preventing sedentary behaviour (SB) should start early in life. Overweight and obesity are caused by a lasting positive energy balance occurring when energy intake outweighs energy expenditure [4]. Consequently, prevention of unnecessary weight gain should target modifiable behaviours that influence energy intake and expenditure including diet, PA and SB.

The school is recognized as an important setting for childhood health promotion interventions since children spend much time at school, and schools have the possibility to implement educational programmes and create a health promoting environment [5,6]. Moreover, reviews have indicated that school-based obesity prevention interventions can be successful [7,8].

Because parents are of major importance regarding their children's nutrition, PA and SB [9-11], parents should be actively involved in school-based obesity prevention efforts [12]. However, to date, no conclusive evidence is available concerning the effectiveness of engaging parents in school-based interventions and what could motivate and enable parents to be engaged in such efforts [13-16]. Hingle et al. [13] and O'Connor et al. [16] reviewed the literature regarding what type of parental involvement was most effective in changing dietary and physical activity outcomes in their children, respectively. School-based interventions that used direct methods to engage parents (e.g. parental education sessions, workshops) were more likely to report positive results compared with those studies that used more indirect methods (e.g. educational information materials, home work assignments). Furthermore, interventions in which parents were engaged via their children were also more likely to have positive findings. Both reviews found that indirect methods were most commonly used to engage parents [13,16]. Nevertheless, in most intervention studies it remains unclear how many and which parents participated. Involving parents in school-based interventions remains challenging since parents are often not eager to participate in school-based interventions and, moreover, they have little spare time next to their work and household [17,18]. Therefore, it is important to identify motivators, facilitators and barriers of parental involvement in school obesity prevention interventions (including both home-and school-based activities), but such studies are lacking.

The 'EuropeaN Energy balance Research to prevent excessive weight Gain among Youth' (ENERGY) project is a multi-center European research project that aims to develop and formatively evaluate a theory-informed and evidence-based multi-component school-based and family-involved intervention programme targeting energy balance-related behaviours (EBRBs) among ten- to twelve-year-old children [19].

The present study is part of the ENERGY project and describes the focus group research that was executed with parents in four countries (Belgium, Hungary, Norway and Spain) to explore what parents' perceptions about parental involvement in school obesity prevention interventions (including both home-and school-based activities) are, including preferred activities, and motivators and barriers of parental participation. In addition, the following research question was also included to explore differences in parent-perceived school health promotion practices between countries: 'what are parents' opinions about general parental involvement and communication, school policy concerning health promotion and the role of schools and parents in obesity prevention'. The focus group research further aimed to obtain the views of parents from different socio-economic classes and with different degree of parental involvement in school activities.

## Methods

The focus group research took place in 4 European countries (Belgium, Norway, Hungary and Spain). By using focus groups, it was possible to elicit a range of perspectives from parents of ten- to twelve-year-old children about parental involvement in school obesity prevention. Focus groups were chosen as a method for several reasons. This focus group research was set up as a first step in the ENERGY intervention development. For convenience purposes, qualitative research was chosen to collect information faster. Moreover, the topics included in the focus groups were non-sensitive and were easily discussed by the participants. The flexible questioning and synergetic effect of group conversations increases the likelihood that data and ideas will be produced that would remain uncovered with other methods (e.g. individual interviews). From the interactions between participants, more insight is often gained into how people think and talk about the topic under investigation. Furthermore, focus groups are valuable to generate ideas about possible effective intervention strategies.

A protocol was developed requiring that each country had to conduct at least four focus group interviews with parents of ten- to twelve-year-olds based on variation in parental socio-economic status (SES) and extent of parental involvement in school activities since family income and education are related to parental involvement [20]. Focus groups were conducted between December 2009 and March 2010. Ethical approval was asked and granted in the different countries by the Ethical Committee of Ghent University (Belgium), the National Scientific Council and Research Ethical Committee (Hungary), and the Ethical committee of the Hospital of Aragón (Spain). Ethical approval of the Regional Medical Ethics committee in Norway was not necessary.

### Participant recruitment

Each study centre except Norway conducted four focus groups. Two of the groups were homogeneous groups: one with parents with high involvement in parent committees and one with parents having a low interest in nutrition, PA and SB in general. A third group was a group with parents from different SES backgrounds and different levels of involvement in school activities. A fourth group consisted of parents from low and medium SES backgrounds as it proved to be impossible to bring together a group with only low SES parents. When representatives of all target groups (high involved, low interest, mixed SES and involvement and low/medium SES) were included in the focus groups, no further groups were recruited. However, the Norwegian study centre only conducted focus groups for the homogeneous groups because they did not managed to recruit low SES and parents with little interest that were willing to participate. The SES of the parents was determined based on parents' educational level. The recruitment of parents occurred mostly through schools and through networks of the researchers. Parents of low SES groups were recruited through schools in deprived city areas or through organizations that work with this specific group. Parents with a low interest in nutrition and PA were recruited through schools and personal contacts and the selection was based on the daily experiences with the parents (e.g. the food brought to the school by the children, the lack of interest in health promotion initiatives). Because of their low interest in health issues it was difficult to motivate these parents to come to a meeting on a fixed day and hour. For this reason also small focus groups were allowed so that we could meet on a convenient moment for these parents. Although these low interest groups do not meet all the criteria for focus groups (e.g. number of participants in focus group), the data were included in the analysis because the perspective of the parents with a low interest in nutrition, PA and SB was particularly important for this study. In some countries (Belgium, Hungary, Norway), incentives (vouchers for toyshop/supermarket, cinema tickets, gift cards) were given.

### Standardization and quality control

To obtain standardization in procedures and conduction across countries, a structural protocol with requirements and methodology was written by the coordinating centre and approved and strictly followed by all other countries. The study protocol was based on established guidelines [21-23] and consisted of detailed practical instructions on topics like recruitment, location and settings, tape equipment and duration of sessions. It also included detailed information for the moderators on how to prepare and lead the focus group discussions. In addition, a semi-structured questioning route was developed and included in the manual to ensure consistency in questions asked across groups (see Additional file 1: Appendix A). Furthermore, a one page demographic questionnaire was filled out by the participants. The questioning route was designed to reveal the actual practices in school-parent contacts and preferences of the parents as recommended in the Intervention Mapping Approach for Programme Design [24]. The content of the questioning route was based on a study of Sy et al. [25] examining the predictors of parental involvement in children's education, and an adaption of the developmental niche theory was used as conceptual framework [26]. This framework suggests that parents' beliefs about education are related to parents' involvement in children's education. In this study, we wanted to apply this to parental involvement in health promotion. The questioning route included the following topics: general communication between schools and parents; parental perceptions about health promotion activities at school regarding nutrition, PA and SB; the role of the school, teachers and parents in health promotion; facilitators and barriers for parental participation in school-based nutrition, PA and SB health promotion; parental perceptions about important health promotion topics that need more attention.

In each study centre, focus group interviews were led by a trained moderator facilitating the group discussions accompanied by a co-moderator taking notes during the sessions. After each focus group session the moderator and co-moderator debriefed by summing up the most interesting discussions, describing the members of the focus group and noting any particular circumstances that might have influenced the discussions. Informed consent which included that study results were handled anonymous and confidential (oral or written) was obtained from all participants.

### Data-analyses

All sessions were audio taped; verbatim transcripts were made in the original language without the names of the participants. Subsequently, a qualitative inductive content analysis of the transcripts was conducted in each country [27]. The different key findings of the focus groups were identified in national summary reports, separate reports were provided for the different type of parents (e.g. high involved, low/medium SES parents). These focus group summary reports were written using a standardized template -based on the key topics of the questionnaire- that was developed for each of the moderators and co-moderators to complete in English. One researcher (WVL) then separately analyzed and summarized the information available from each of the four summary reports. The integrated results from the four study centers were validated by the (co-)moderators (MB, NL, JMFA, EK) of all participating countries.

## Results

The results of the focus groups are presented below starting with a short introduction on parents' perceptions about general parental involvement and health promotion initiatives at school, followed by the key themes of this article namely the role of school, parents, others; preferred health promotion activities; and facilitating factors and barriers of parental participation in school-based interventions. The findings are presented separately for nutrition, PA and SB.

### Participants

Seventeen focus group interviews were conducted with a total of 92 parents (12 men, 80 women; age = 41.1 ± 6.4 years; number of children = 2.2 ± 0.9), the number of participants per focus group ranged from two to ten.

In general, no clear differences between low/medium SES, high-involved, and non-interested parents were found. When dissimilarities occurred, this was stated explicitly.

**Figure 1:**
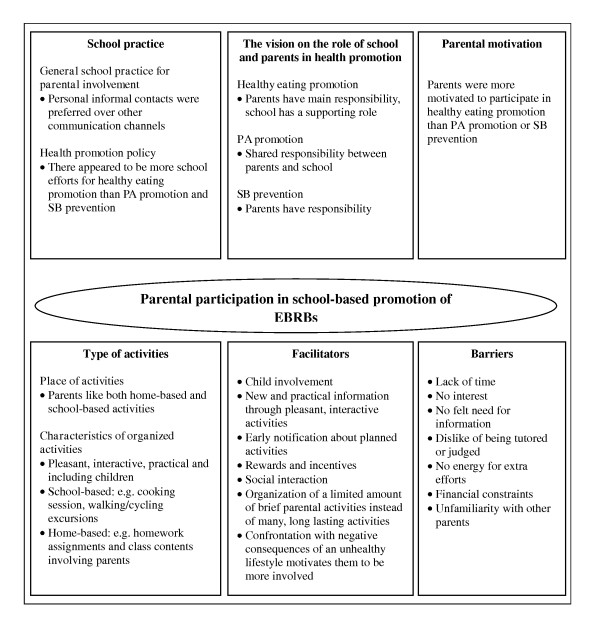
**Factors related to parental involvement in EBRBs (parents' perspective) and recommendations for interventions based on focus groups with parents in 4 European countries**.

Table 1 gives an overview of the focus groups conducted for this study. In Figure 1 an overview is given of the vision of parents on the health promotion policy in schools, the role of the school and the parents in EBRBs promotion, the barriers and facilitating factors for parental involvement in EBRB promotion, and the parents' preferences for the type of activity.

**Table 1:** Overview of the focus group participants.

	Gender (%women)	Age range	SES	N° of participants per group
Focus group 1 (high involved)	87%	35-53	Medium-high	4-7

Focus group 2 (low interest)	81%	29-55	Low-medium	2-6

Focus group 3 (mixed SES/involvement)	85%	35-49	Medium-high	8-10

Focus group 4 (low/medium SES)	95%	25-47	Low-medium	5-7

The table included an overview of the demographics of the participating parents across the countries in the different focus groups

### Parental perceptions about general parental involvement and health promotion at school

In two of the four countries (Belgium, Spain), parents reported to be satisfied with the communication between the school and the parents, while Hungarian and Norwegian parents mentioned being less satisfied with the information flow between school and parents. Parents discussed that face-to-face contacts and written communication through letters, website and school diary were mostly used as communication channels. Most parents indicated to have a clear preference for informal personal contact and mentioned that this is the best way to have a good communication and information transfer.

#### Promotion of healthy eating at school

Across all countries, parents mentioned being aware of the organization of many initiatives to promote a healthy diet including availability of free school fruit, fruit competitions (the child bringing the most fruit pieces per week to school wins), provision of educational materials, nutrition education, nutrition projects, and rules and restrictions of unhealthy foods. However, some parents in all countries indicated that more efforts could still be made.

#### Promotion of physical activity and prevention of sedentary behaviour

In most countries (Hungary, Norway and Spain) only few initiatives were mentioned by the parents. In Belgium, a lot of initiatives to promote PA were undertaken by the school according to the parents. Nevertheless, parents in all countries indicated wanting more PA opportunities next to the mandatory physical education classes and, moreover, they asked for non-competitive PA initiatives. Promoting PA through active transport appeared to be a difficult issue in most countries because of dangerous traffic, lack of bike storage, or risk for theft. None of the parents mentioned initiatives to prevent excessive screen time use. Moreover, a group of parents thought that schools could not influence this behaviour (Belgium, Spain).

### Role of school, teachers, and parents in promoting EBRBs

#### Promotion of healthy eating

In all countries, parents indicated that both schools and parents are responsible for the promotion of a healthy diet. However, parents' opinions about who has the main responsibility were mixed. Most parents (Belgium, Norway, and half of Hungarian and Spanish parents) pointed out being most accountable for their children's diet but mentioned that they need to be supported by the school as children spend a lot of their time at school. If not, parents' emphasis on healthy nutrition will be counteracted by the school. Other parents (Belgium, Hungary, and Spain) highlighted the importance of teachers and schools in raising consciousness about healthy eating in children. Parents mentioned that teachers are often seen as role models by the children and might therefore have a substantial influence on children's behaviours. Some Hungarian and Spanish parents thought that school and parents have a shared responsibility in this.

A small group of parents also mentioned other organizations that need to take their responsibility such as youth organizations and sport clubs (Belgium, Norway). Some parents (Belgium, Hungary, and Norway) complained about the role of media, ads and marketing because they find it very difficult to counteract or balance these influences.

#### Promotion of physical activity

All parents mentioned that parents and schools have a shared responsibility for promoting PA but some parents (all Belgian, and some Spanish and Hungarian parents) attributed the main responsibility to the parents with a supporting role of the school. According to them, parents are in the best position to influence their children's PA because they can stimulate and motivate them to be active after school hours or in the weekend. However, parents also mentioned that school initiatives are influential because children spend much time at school. A small group of parents (Hungary and Spain) indicated that PA is not the first priority, learning good manners, prevention of addictions (Hungary) and nutrition education (Spain) are more important. Only Hungarian parents reported that also other organizations need to stimulate more PA. According to them, the municipality should organize non-competitive mass PA events for all children and needs to improve the PA environment in the community (safety and quality). In addition to this, sport clubs should be less competitive-oriented.

#### Prevention of sedentary behaviours

In most countries (Belgium, Norway, Spain), parents mentioned that SB is an issue of the family. Belgian parents mentioned that the school has no effect on this behaviour and therefore efforts are useless, moreover, schools seem to have a negative effect on screen time use (e.g. by giving assignments for computer tasks or watching certain programmes). Some Norwegian parents indicated that they would have liked the schools to agree on common rules for all children in a class/group/neighborhood, but they know that this is not realistic.

### Motivation to participate in school-based interventions

#### Promotion of healthy eating

Most Belgian and Spanish parents indicated to be willing to participate in healthy nutrition promoting activities whereas most Hungarian and Norwegian parents reported not wanting to be involved in these activities.

#### Promotion of physical activity and prevention of sedentary behaviour

A majority of the parents (Belgium, Hungary, and Norway) mentioned being not motivated to participate in activities to promote PA and decrease SB. In contrast, most Spanish parents indicated to be enthusiastic about these initiatives.

### Type of preferred health promotion activities

#### Promotion of healthy eating

To involve parents, both school- and home-based activities were mentioned as good options.

##### School-based activities

Parents indicated that providing information through theoretical lectures at parental evenings is not attractive but boring and few parents will participate (Belgium, Spain). However, a small group of parents found lectures by experts interesting (Belgian low/medium SES and Norwegian high involved group). Parental events need to include pleasant, interactive, useful, practical, and informative activities, and also involve children according to parents in all countries except for Norwegian parents who mentioned only few parental activities. School events that parents particularly like were: a healthy breakfast, a 'healthy cooking' workshop, and a 'how to buy' workshop. Tailored nutrition counseling sessions were mentioned in a negative way by some parents (Belgium, Hungary), this type of activity was considered as interfering with the privacy of the family.

##### Home-based activities

All Belgian and a few Spanish and Hungarian parents gave examples of favorable home-based activities. Home work activities (e.g. visit a local supermarket, make a healthy snack) with active parental involvement, were mentioned as an option but parents clearly stated that these assignments need to be pleasant, practical and interesting. Educational materials (e.g. brochures, leaflets) were mentioned by some parents as another possibility to engage parents (Belgium, Hungary) but opinions about the effectiveness of this strategy were mixed, these parents think only parents already interested in nutrition will read these materials. Nevertheless, parents mentioned that written materials need to be attractive, focus on both child and parent and include useful and practical information (e.g. recipes, tests, and tips), a website could be a good alternative for this. High involved parents in Hungary and Belgium suggested class contests involving parents (e.g. cooking competition for best recipe) to boost parental involvement.

#### Promotion of physical activity and prevention of sedentary behaviour

Solely Spanish parents showed high motivation to participate in school-related PA promotion and prevention of SB. Therefore, it is no surprise that only a few preferred activities were mentioned across all countries. Moreover, no initiatives concerning the prevention of excessive SB were cited.

##### School-based activities

Across all countries except Norway, practical school events were mostly mentioned. A majority of parents (Belgium, Hungary, and Spain) indicated preferring the organization of fun activities of moderate intensity together with their children instead of sport activities. Given examples were walking excursions, cycle tours, and survival days. Hungarian parents also mentioned these activities but seemed not very eager to participate themselves. A small group of parents (Belgian and Spanish high involved and Belgian low/medium SES) indicated that they would like sport activities such as how to start to run/swim activities, football or basket-ball competitions. In addition, parental events where information about possible sport possibilities in the neighborhood is presented and/or the importance of PA is given, were mentioned by most Belgian parents. However, these events need to be attractive, pleasant, active and interactive. Another possibility to include parents in PA promotion is the organization of active transport to school through walk or cycle pools with parents, however, almost all parents except Norwegian parents mentioned that this is a bad idea because of safety issues, weather, lack of bicycle parking, and/or long distance from home to school.

##### Home-based activities

Only Belgian and Hungarian parents provided concrete examples of home-activities they like. Educational materials (e.g. newsletter, brochures, leaflets) including information about health effects of PA, PA opportunities in the community, and guidelines for PA were considered as interesting by half of those parents. However, some parents think only highly interested parents would read this and therefore it would be useless, a good alternative could be a website. Nevertheless, parents stated that educational materials should be useful, practical, funny and focus on children's behaviour. Other mentioned activities to motivate parental participation were: a PA contest (e.g. rewards for the most active class) and home work assignments about PA (Belgium). Some Hungarian parents mentioned that they also would like community activities. Norwegian parents did not mention any preferred activities but they stated that they would like to learn more about ways, tools and possibilities to motivate their children to be active.

### Facilitating factors of parental participation in school-based intervention activities

#### Promotion of healthy eating

A majority of parents (Belgium, Spain, Hungary) stated that child involvement in parental activities is very important, sensitization and motivation of parents only succeeds through children. Parents also mentioned the importance of organizing practical and interactive activities providing new and interesting nutrition information. Half of the Belgian parents (high involved and low/medium SES) mentioned being more motivated to participate when only a few, brief school activities are organized.

Both home- and school-related activities were discussed, but no real preference was expressed. Some Belgian parents (low/medium SES) thought children would be more enthusiastic about school-based activities together with their friends, parents' experience this as important since involving children is of key importance for them. According to the parents, an asset of home-based activities was that parents can plan these at a convenient moment. Nevertheless, information about time and date of planned activities beforehand is a facilitating factor for both home-and school-based activities. Parents indicated being more motivated to participate if they could plan this carefully some time in advance (Belgium). According to Belgian high-involved parents, another asset of home-related activities, especially homework assignments, is that non-native parents are more likely to be reached since children and parents can use their native language to conduct the homework assignments.

Some Belgian parents (high involved) stated that confronting them with the negative consequences of an unhealthy diet would convince them to be more involved in nutrition activities. Finally, economic motives were also mentioned by some parents (Belgium, and Hungary). Several Belgian parents thought that some parents would be more eager to participate when receiving a reward or incentive. Hungarian parents confirmed the importance of rewards by mentioning the provision of free activities or healthy lunch boxes. Some Belgian parents (low interest) mentioned the importance of democratic prices for activities organized for parents. None of the Norwegian parents gave possible facilitators for parental participation.

#### Promotion of physical activity and sedentary behaviour

Similar with participation in nutrition activities, child involvement was seen as fundamental to encourage parents to participate according to all Belgian and some Hungarian (high involved) and Spanish (low/medium SES and low interest) parents. Moreover, some parents (Belgium) highlighted that activities need to focus on children instead of parents. Parents mentioned being more motivated to participate when organized activities are fun, practical and useful (Belgium, Hungary). As with healthy eating activities, a group of parents (Belgium) indicated that being informed about planned activities beforehand may increase willingness to participate. Other facilitating factors mentioned by some parents were economic incentives e.g. financial support for PA or organization of financially affordable activities (Belgium, Hungary) and social contact with other parents (Spanish low/medium SES and high involved Hungarian parents).

### Barriers of parental participation in school-based intervention activities

#### Promotion of healthy eating

The top reason reported for non-participation was the lack of time. All Belgian and Norwegian and half of Hungarian parents mentioned that it is difficult to find a convenient moment for these kinds of activities. In contrast, only Spanish low interest parents gave this as an explanation. A majority of the parents indicated having no interest in nutrition (Belgium, Hungary) or already having enough knowledge as a result of nutrition information overload (Belgium, Hungary, Spain, Norway), as motives for non-participation. Some parents mentioned that parents of obese children might feel stigmatized when taking part in school activities and this could be a barrier. Other barriers mentioned were: dislike of being tutored or judged, no energy for extra efforts on top of normal daily activities and duties (Belgium), and financial constraints related to the extra costs for healthy foods (Hungary).

#### Promotion of physical activity and prevention of sedentary behaviour

The most important barrier mentioned for participation was again time constraint. Parents mentioned having a busy life (e.g. demanding job, running household), so they had difficulties to find a convenient moment for such activities. Several parents (Spain, Belgium, Hungary) also mentioned that children prefer activities with their peers instead of their parents. Moreover, some parents (Spain) indicated the lack of common interests between parents and children as a hindering factor. Other mentioned reasons for non-participation were: no interest/motivation (Hungary, Spain), bad weather for PA activities (Belgium), already having enough knowledge (Belgium, Hungary), and not wanted to be tutored by teachers/school (Norway, Spain). Some Hungarian parents (low/medium SES and low interest) mentioned money issues as a barrier to promote PA within their children. Spanish low interest parents mentioned unfamiliarity with other parents as an obstacle.

## Discussion

This article presents findings from focus group research related to parental involvement in obesity prevention interventions through the school. Given the differences in cultural and environmental characteristics between the four participating countries, the focus group research aimed at describing preferred activities and important motivators, facilitators and barriers of parental participation across the different countries. Additionally, information was gathered about parental perceptions regarding general school policies for parental involvement and health promotion, as well as parents' opinions about the role of parents and schools in obesity prevention.

Per country, different focus groups with variation in SES and parental involvement and interest in obesity prevention were organized to have a representation of all parents. No clear differences in opinions and preferences were found between parents with different SES or involvement in school activities indicating a general agreement on the issues among all parents. It is possible that the lack of homogeneous low SES groups hindered the differentiation of the perspectives of low in contrast to middle and high SES groups. When comparing overall findings across countries, it appeared that the parent respondents in Hungary and Norway reacted more negative about parental participation in nutrition-, PA-, and SB-related activities than Belgian and Spanish parents. Remarkably, these same Hungarian and Norwegian parents voiced negative feelings with the current general school policy for parental involvement.

Parents had different expectations for how the school should interact with them and their child for the different EBRBs. PA promotion was seen as a shared responsibility between parents and schools whereas healthy eating promotion was mainly considered as a task of parents with support of the school. In contrast, parents stated that SB prevention is solely a family issue in which the school can or should not interfere. Reasons for this belief were not further explored in this study but it has been reported by others that parents do not consider their children's screen time behaviour as excessive or problematic [28], a lack of awareness might play a role in their reluctance to accept school initiatives for this behaviour. Moreover, parents indicated being much more motivated to participate in nutrition promoting activities compared to PA- and SB-initiatives. These findings are in line with previous focus group research, Hart et al. [29] found that parents of seven to twelve-year-old children accepted their role of providing healthy diet for their children but they were less motivated to accept their responsibility for PA promotion. However, as parenting practices are associated with children's PA and SB [30,31], future interventions should aim at enhancing the awareness of the parents' role and support them in their efforts to enhance their child's PA and decrease SB.

Parents gave several indications that the format of the activities for the parents was very important: strategies to engage parents should include practical, interactive, and pleasant activities together with the child instead of purely providing theoretical educational information. Also Perry et al. [32] and Blanchette & Brug [33] acknowledged that child-parent activities -preferably at home- have the best chance to engage parents.

Parents indicated preferring nutrition 'workshop' activities at school instead of theoretical lectures and tailored nutrition counseling sessions. Parents also mentioned that PA promoting initiatives should be practical, fun, of moderate intensity, and for both parent and child. However, active transportation via walking/bicycling pools with parents was considered as a bad idea by most parents for many reasons. Home-based nutrition and PA activities (e.g. home work assignments) were considered a good alternative for school initiatives to engage parents. In contrast, the usefulness of educational materials should be given careful considerations as parents' enthusiasm and interest was mixed. Parents highlighted that in case educational materials will be provided (e.g. leaflet, brochure), great efforts should be made to make these as attractive as possible. This is in line with a study of Crockett et al. [34], in which parents indicated that they like home-based nutrition interventions (e.g. worksheets, homework and activities to do with their child at home) and do not like parent group meetings.

Noteworthy, when comparing the parent's perspectives concerning parental involvement in obesity prevention at school with the six evidence-based types of parental involvement in children's academic achievements identified by Epstein [35,36], parents' preferences for parental involvement activities could be fitted into four of these types: 1) Parenting (e.g. establishing a home environment that support children to eat healthy, to increase PA and decrease SB by increasing knowledge and awareness through several activities), 2) Communicating (e.g. parents prefer face-to-face, interactive activities), 3) Learning at home (e.g. parental involvement in homework about EBRBs), 4) Collaborating with the community (e.g. parents indicated that other organizations in the community should be involved in school-based obesity prevention). However, two of the six major types of parental involvement [35,36] could not be extracted from the parents' responses: 5) Volunteering (e.g. help and support for school functions and activities) and 6) Decision making (participation in making school decisions).

The present study identified numerous potential facilitators and barriers of parental participation in school-based interventions, which should be kept in mind when developing a new intervention. Child involvement was seen as the most important facilitating factor for parental participation in both nutrition and PA promotion activities, this significance of child inclusion is supported by findings from previous studies [34,37,38]. The most often mentioned barrier for parental participation was the lack of time since parents have many obligations (e.g. work, household). Therefore, parents emphasized the importance of timely information about activities for planning purposes A systematic review of qualitative research concerning parental perceptions regarding obesity prevention [39] also identified most of the barriers mentioned by the parents in this study [39]. The identified facilitators for parental involvement provided suggestions for solutions for some of the barriers. Involving children can possibly motivate less interested parents to attend certain parental activities to please their children. By organizing fun activities for both children and parents, children's preference for activities with peers and lack of common interest in PA might also be solved. Rewards, incentives or democratic prices for activities could solve some of the financial constraints mentioned by parents. Some parents were confident about their knowledge of a healthy diet and were therefore less eager to participate. However, it is possible that they underestimate their informational needs concerning healthy eating since this is a common phenomenon amongst European adults [40]. Based on the parents' reluctance against being tutored by the school, health promoters and schools should avoid prescriptive teaching to parents and instead use less hierarchical methods to inform parents. The Self-Determination theory [41] provides a good framework for the development of activities that supports the autonomy of the parents, enhances the development of intrinsic motivation to support their child in healthy behavior and to create less controlling but more supporting environments.

As there are no quantitative studies assessing parents' opinions about parental participation in EBRB-related interventions, this qualitative study is the first to reveal topics that should be further explored and evaluated. A strength is that the focus groups were conducted in four different European countries. The advantage of a focus group format is that it generates a lot of dialogue and discussions and exposes processes that would remain uncovered otherwise. However, a disadvantage of this method is that some parents give social desirable answers. Limitations of this study were the failure to form focus groups with only low SES parents despite our efforts to engage these parents, as well as the low number of participants in some focus groups involving parents with little interest. Another limitation was that mainly maternal views of parental involvement were gathered since only 12 out of the 92 participants were men (13%). The results of this research are also not generally representative for each of the countries because participants were not randomly selected. The aim of this study was to gain more insights in the opinions of parents concerning parental participation in healthy nutrition, and PA promotion and SB prevention. Therefore, representative samples of participants were less important. Because of the difference in languages between the countries, different moderators and co-moderators conducted the focus group research. Consequently, differences in interview style and experience could have influenced the flow and content of the interviews. In order to limit these differences, a well-structured questioning route was used in all focus group interviews.

## Conclusions

Based on this study, the following conclusions could be drawn. However, given the limitations of the recruitment in this study, these findings are not generally representative for all European countries. The parental module for a school-based obesity prevention programme should be practical, pleasant and interesting. Children should be regarded as a stimulating intermediate between school and parents; a direct focus on parents and their behaviour is not preferred. Parents appear to be more interested in nutrition than in PA and SB, therefore, it could be a solution to combine health promotion activities for these EBRBs. Nevertheless, it will be demanding to involve parents. The biggest challenge will be to find a convenient moment for all parents, and to enhance the motivation of groups of parents to be involved in promoting EBRBs.

## Competing interests

The authors declare that they have no competing interests.

## Authors' contributions

WVL, MV, IDB and LM developed the standardized protocol and the semi-structured questionnaire. WVL, MV, NL, MB, EK, and JMFA conducted the focus group research in the different countries which included conducting the interviews, transcribing the audiotapes, conducting qualitative content analysis on the transcripts, and summarizing into national report). WVL wrote the manuscript. All authors were involved in drafting the article and revising it critically for important intellectual content. The content of this article reflects only the authors' views and the European Community is not liable for any use that maybe made of the information contained therein.

## Pre-publication history

The pre-publication history for this paper can be accessed here:

http://www.biomedcentral.com/1471-2458/11/881/prepub

## Supplementary Material

Additional file 1 **Semi-structured questioning route**. This file contains the semi-structured questioning route that is used to conduct the focus group interviews across all countries. Click here for file
