# Supplementary material for: What do parents think about parental participation in school-based interventions on energy balance-related behaviours? a qualitative study in 4 countries
Source: BMC Public Health. 2011 Nov 23;11:881. doi: 10.1186/1471-2458-11-881 (PMC3252293; doi:10.1186/1471-2458-11-881)
Supplement: Additional file 1 — Semi-structured questioning route. This file contains the semi-structured questioning route that is used to conduct the focus group interviews across all countries. [file 1471-2458-11-881-S1.DOC]

Appendix A

**Satisfaction with general school policy parental involvement / health promotion policy**

1. Are you satisfied with the contact the school of your child has with the parents?

2. Which health eating and PA promoting activities are organized by the school?

**Role of the school in healthy nutrition and PA promotion, and SB prevention**

3a. Is healthy eating an issue of the home, the school, both or should others take care of this issue?

3b.Is physical activity and TV viewing an issue of the home, the school, both or should others take care of this issue?

**Motivation in healthy nutrition and PA promotion, and SB prevention**

4a. Would you like to be involved in school activities concerning health eating?

4b. Would you like to be involved in school activities concerning sports and physical activity?

**Methods for parent involvement / facilitators and barriers in healthy nutrition and PA promotion and SB prevention**

5. Would you like that the school does the following for the parents:

Organizing educational events, practical events, counseling, other

Why do you prefer one or more of these types of activities?

Why do you not like some of these activities?

6. Would you participate yourself in these activities?

Which ones?

Why?

Why not?

In which activities would you certainly not want to participate?

7. Are there other activities that you think should be organized?

8. What can be done to increase parental involvement in activities at school?

9. What would motivate you to participate?
